# Induction of Apurinic Endonuclease 1 Overexpression by Endoplasmic Reticulum Stress in Hepatoma Cells

**DOI:** 10.3390/ijms150712442

**Published:** 2014-07-14

**Authors:** Tsung-Lin Cheng, Pin-Shern Chen, Ren-Hao Li, Shyng-Shiou Yuan, Ih-Jen Su, Jui-Hsiang Hung

**Affiliations:** 1 Department of Physiology, College of Medicine, Kaohsiung Medical University, Kaohsiung 80708, Taiwan; E-Mail: junglecc@kmu.edu.tw; 2 Department of Biotechnology, Chia Nan University of Pharmacy and Science, Tainan 71710, Taiwan; E-Mails: pschen@mail.chna.edu.tw (P.-S.C.); spadem39445@gmail.com (R.-H.L.); 3 Translational Research Center, Cancer Center, Department of Medical Research, and Department of Obstetrics and Gynecology, Kaohsiung Medical University Hospital, Kaohsiung Medical University, Kaohsiung 80708, Taiwan; E-Mail: yuanssf@ms33.hinet.net; 4 National Institute of Infectious Diseases and Vaccinology, National Health Research Institutes, Tainan 70456, Taiwan; E-Mail: suihjen@nhri.org.tw; 5 Drug Discovery and Development Center, Chia Nan University of Pharmacy and Science, Tainan 71710, Taiwan

**Keywords:** hepatocellular carcinoma, Huh-7, HepG2, NeHepLxHT, hepatitis B virus, hepatitis B virus pre-S2∆ large mutant surface protein, endoplasmic reticulum stress, apurinic endonuclease 1, GRP78

## Abstract

Hepatocellular carcinoma (HCC) is one of the most common malignancies worldwide with poor prognosis due to resistance to conventional chemotherapy and limited efficacy of radiotherapy. Previous studies have noted the induction of endoplasmic reticulum stress or apurinic endonuclease 1 (APE1) expression in many tumors. Therefore, the aim of this study was to investigate the relationship between endoplasmic reticulum (ER stress) and APE1 in hepatocellular carcinoma. Here we investigate the expression of APE1 during ER stress in HepG2 and Huh-7 cell lines. Tunicamycin or brefeldin A, two ER stress inducers, increased APE1 and GRP78, an ER stress marker, expression in HepG2 and Huh-7 cells. Induction of APE1 expression was observed through transcription level in response to ER stress. APE1 nuclear localization during ER stress was determined using immunofluorescence assays in HepG2 cells. Furthermore, expression of Hepatitis B virus pre-S2∆ large mutant surface protein (pre-S2∆), an ER stress-induced protein, also increased GRP78 and APE1 expression in the normal hepatocyte NeHepLxHT cell line. Similarly, tumor samples showed higher expression of APE1 in ER stress-correlated liver cancer tissue *in vivo*. Our results demonstrate that ER stress and HBV pre-S2∆ increased APE1 expression, which may play an important role in resistance to chemotherapeutic agents or tumor development. Therefore, these data provide an important chemotherapeutic strategy in ER stress and HBV pre-S2∆-associated tumors.

## 1. Introduction

Hepatocellular carcinoma (HCC) is the fifth most common cancer and the second leading cause of cancer death in men worldwide [1]. Hepatocellular neoplasms develop regularly from preneoplastic foci of altered hepatocytes, and hepatocellular cancer occurs both sporadically and in relation to chronic viral infection [2], environmental exposure [3], extensive alcohol intake [4], transgenic oncogenes [5,6] and alternative causes of hepatic cirrhosis. Globally, hepatitis B virus (HBV) accounts for 53% of all cases of HCC [7]; chronic HBV infection is one of the major causes of HCC, and it is estimated that over 350 million people are chronically infected with HBV worldwide [8,9]. HBV encodes three envelope proteins in the pre-S/S open reading frame that are named large, middle, and small surface proteins. One of the major mutant types is caused by the deletion of the pre-S2 region (pre-S2Δ). These pre-S2Δ mutants are becoming increasingly prevalent in serum and liver tissues of patients with chronic HBV infection and HCC [10,11,12,13].

Apurinic apyrimidinic endonuclease redox effector-1 (APE1/Ref-1) is a protein involved in the regulation of gene expression as a redox co-activator of different transcription factors and in the base excision repair (BER) pathways of DNA lesions [14,15,16]. APE1 belongs to a large family of nucleases and play an important role in DNA repair, initiating the elimination of AP sites. AP sites arise in DNA when the *N*-glycoside bond is hydrolyzed and a damaged base is excised by DNA glycosylases. On the other hand, as a redox factor (Ref-1), APE1 regulates gene expression by activating transcription factors such as Jun, Fos, p53, Early growth response protein-1 (Egr-1) and NF-κB [17,18]. These different activities are located in two functionally distinct domains: The *N*-terminus is principally devoted to the redox activity while the *C*-terminus exerts the enzymatic activity on the DNA bases [19]. Recent studies have indicated that APE1 showed high expression in a variety of cancers including germ cell tumors, gliomas rhabdomosarcomas, breast, liver, non-small cell lung cancer and ovarian cancer [20,21,22,23,24,25]. In addition, overexpression of APE1 has been correlated with poor outcome to chemoradiotherapy [26,27]. Therefore, modification of APE1 redox function or DNA repair activity may be an important target for chemotherapeutic drugs.

The endoplasmic reticulum (ER) is a dynamic membranous organelle that plays an important role in protein folding, transport, and processing. Unfolded proteins in the endoplasmic reticulum activate several signaling pathways that are referred to as the unfolded protein responses (UPR). The UPR pathway has three components in mammalian cells: basic leucine zipper transcription factor ATF6, IRE1 RNA-processing enzyme, and protein kinase-like ER kinase (PERK). Endoplasmic reticulum stress (ER stress)-mediated activation of the UPR occurs in cancer tissues and is involved in the regulation of gene expression in cancer cells [28,29,30]. Many chemical agents, viral proteins, and adverse metabolic conditions cause protein misfolding or protein accumulation in the ER, leading to ER stress. Research over the past decade has also demonstrated that many physiological conditions cause ER stress, e.g., nutrient or glucose deprivation, degenerative neuronal disorders [31,32], diabetes [33], differentiation of B-cells into plasma cells [34], and virus infection [35,36]. Furthermore, our previous studies have shown that overexpression of pre-S2Δ large surface proteins are observed in the induction of ER stress [37,38]. Based on epidemiologic studies, HBV carriers who presented with the pre-S2Δ mutant protein in serum had worse disease outcomes than those who did not [39]. Therefore, the aim of this study was to assess the relationship between APE1 expression and ER stress in hepatocellular carcinoma cells *in vitro* and *in vivo*, and the possible role of APE1 in HepG2 cells in response to ER stress.

## 2. Results and Discussion

### 2.1. Induction of Apurinic Endonuclease 1 (APE1) Expression by Endoplasmic Reticulum (ER) Stress

**Figure 1:**
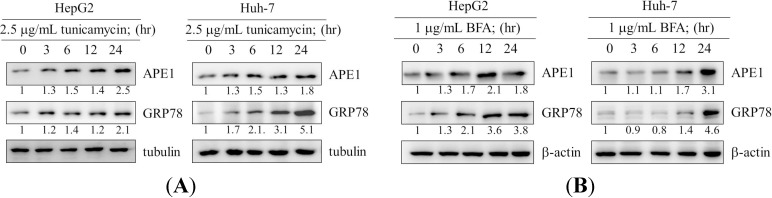
Elevated expression of APE1 in response to endoplasmic reticulum stress. (**A**) and (**B**) present the time- dependent effect of ER stress inducer, tunicamycin and brefeldin A, on APE1 expression. HepG2 and Hun-7 Cells were exposed to tunicamycin and brefeldin A in 10% FBS-supplemented DMEM for the times indicated. The cell lysates were analyzed by western blotting with antibodies for APE1, GRP78, tubulin, and β-actin. The expression levels of APE1 and GRP78 were quantified by using ImageJ software (National Institutes of Health, Bethesda, MD, USA).

Huh-7 and HepG2 human hepatocellular carcinoma cells were treated with tunicamycin or brefeldin A (Figure 1A,B). When HepG2 and Huh-7 cells were incubated with tunicamycin or brefeldin A, an increase in the level of APE1 was observed in a time-dependent manner. The level of APE1 protein was induced at 6–24 h after treatment with tunicamycin (Figure 1A), and the APE1 level at 24 h was approximately 3-fold higher than that expressed at 0 h. Similarly, using another ER stress inducer, brefeldin A, APE1 protein was significantly induced with time up to 24 h. For example, the APE1 level at 24 h was enhanced up to about 4-fold compared with 0 h (Figure 1B). Expression of GRP78 protein was used to check that treatments in our system indeed resulted in ER stress.

### 2.2. Induction of APE1 Expression Is Transcription-Dependent

**Figure 2:**
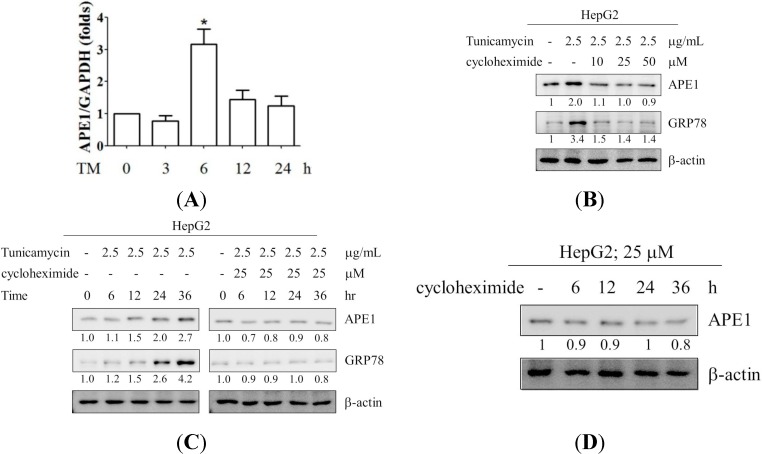
Induction of APE1 mRNA expression by tunicamycin (TM) was determined by real-time PCR in HepG2 cells (**A**). The cells were exposed with 2.5 μg/mL tunicamycin for times indicated. The total RNA was isolated and then subjected to real-time PCR analysis. Real-time PCR was performed as described in the Experimental Section. *Columns*, mean of three independent experiments; bars, SD (*, *p* < 0.05, Student’s *t* test); (**B**,**C**) Induction of APE1 expression by tunicamycin was inhibited by CHX in HepG2 cells. The cells were exposed with 2.5 μg/mL tunicamycin in the presence of CHX for as dose and time-indicated. The expression of APE1, GRP78 and β-actin were analyzed by Western blotting. The expression levels of APE1 and GRP78 were quantified by using ImageJ software; (**D**) HepG2 cell were treated with CHX for 6, 12, 24, and 36 h. The cell lysates were analyzed by western blotting with antibodies for APE1 and β-actin. The expression level of APE1 was quantified by using ImageJ software.

To establish the mechanism of APE1 expression by ER stress, APE1 mRNA expression was evaluated during ER stress. HepG2 cells were treated with tunicamycin for the times indicated (Figure 2A). APE1 mRNA expression level was increased in response to ER stress in HepG2 cells. In addition, to block *de novo* protein synthesis, cycloheximide (CHX) was used. When HepG2 cells were co-treated with CHX and tunicamycin for the dose and time indicated, we found that expression of APE1 was significantly inhibited by CHX in a dose and time-dependent manner (Figure 2B,C). Furthermore, we also evaluated the effects of CHX on expression status of APE1 in HepG2 cells. The cells were incubated with CHX for 0, 6, 12, 24, 36 h, and APE1 expression was analyzed. The expression of APE1 was decreased by CHX treatment. The data suggested that the elevated expression of APE1 protein during ER stress is contributed by increasing the expression of APE1 transcripts.

### 2.3. Nuclear Localization of APE1 during ER Stress

**Figure 3:**
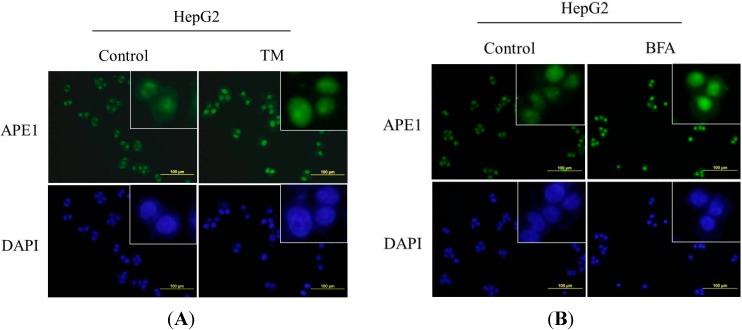
Nuclear localization of APE1 during ER stress. (**A**,**B**) Nuclear localization of APE1 in response to ER stress in HepG2 cells. The cells were treated with 2.5 μg/mL tunicamycin or 1 μg/mL BFA, and the localization of APE1 was determined by using immunofluorescence staining. Scale bar: 100 μm.

The main function of APE1 protein is its role in DNA repair or as a redox co-activator of different transcription factors that regulate target genes to mediate cellular activities. APE1/Ref-1 subcellular localization is mainly nuclear, but cytoplasmic staining has also been reported, the latter being associated with mitochondria and/or presence within the endoplasmic reticulum [18]. Therefore, we next investigated the distribution of APE1 by immunofluorescence in HepG2 cells following the treatment of ER stress inducers. As indicated in Figure 3A,B, little APE1 was detected in the untreated cells; however, the APE1 signal was significantly enhanced and distributed exclusively in nuclei in the tunicamycin or brefeldin A-treated cells.

### 2.4. Downregulation of APE1 Expression Reduced Cell Survival in Response to ER Stress

**Figure 4:**
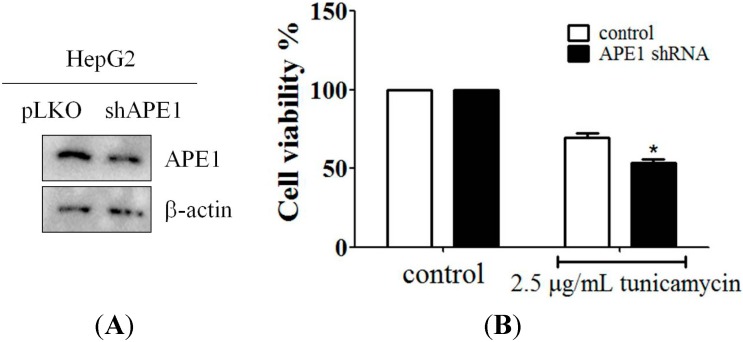
APE1 was involved in tunicamycin-induced cell death. (**A**) Down-regulation of APE1 expression by APE1 shRNA during ER stress. HepG2 cells were transfected with pLKO-APE1 shRNA plasmid, and then expression of APE1 was analyzed by immunoblotting; and (**B**) shRNA-mediated knockdown of APE1 increased HepG2 cells from tunicamycin-induced cell death. The cells were incubated with tunicamycin in 10% FBS-supplemented DMEM for 48 h. Cell viability was measured by the MTT assay. Columns, mean of three independent experiments; bars, SD (*, *p* < 0.05, Student’s *t* test).

In order to confirm the importance of APE1 expression in ER stress, the pLKO-APE1 shRNA vector was transfected into HepG2 cells to knockdown APE1 expression. The efficiency of *APE1* gene silencing was evaluated by using western blotting (Figure 4A). Next, an MTT assay was applied to measure cell survival during ER stress in HepG2 cells, and the result indicated that knockdown of APE1 expression was increased cell survival from tunicamycin-triggered cell death (Figure 4B), thereby indicating that APE1 is indispensable in the process of ER stress-induced cell death.

### 2.5. Hepatitis B Virus Mutant Large Surface Protein Can Induce APE1 Expression in Vitro and in Vivo

**Figure 5:**
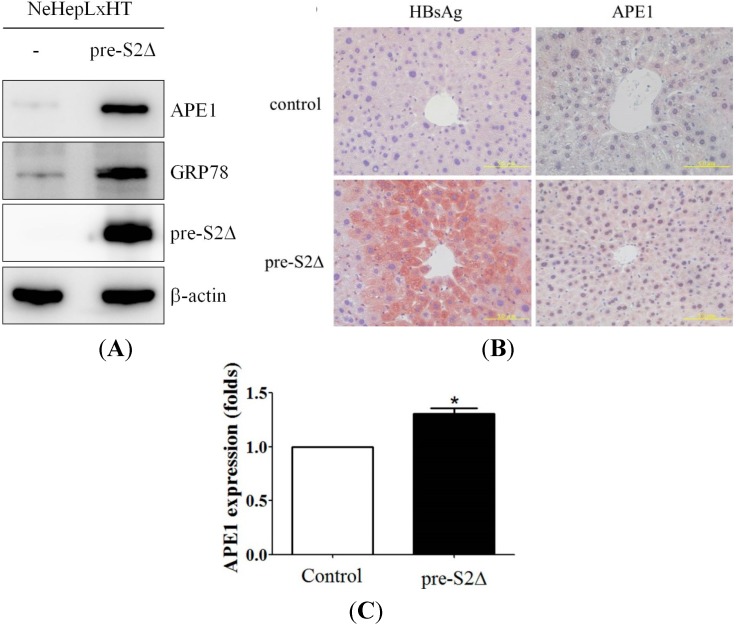
Induction of APE1 expression by HBV large surface mutant protein *in vitro* and *in vivo*. (**A**) APE1 expression was induced by ER stress-associated protein, pre-S2Δ, *in vitro*. The NeHepLxHT cells and NeHepLxHT-pre-S2Δ cells were cultured in 10% FBS-supplemented DMEM. The total cell lysates were analyzed by Western blotting with antibodies for APE1, GRP78, and β-actin; (**B**) Elevated APE1 is associated with pre-S2∆ expression *in vivo*. The expression of APE1 and HBV surface proteins in liver tissues of control and pre-S2∆ transgenic mice was analyzed by immunohistochemical staining (×400). Scale bar: 50 μm; and (**C**) The expression levels of APE1 were quantified by using ImageJ software. The data represent the mean of APE1 protein expression level from three independent experiments. Columns, mean of three independent experiments; bars, SD (*, *p* < 0.05, Student’s *t* test).

ER stress can be induced by either drugs such as tunicamycin or by overexpression of mutant proteins including virus gene products. From our previous study, hepatitis B virus mutant large surface proteins have been shown to induce ER stress [35,37]. Therefore, it is important to investigate whether the mutant large surface protein can induce the expression of APE1. The expressions of APE1 and GRP78 were examined in the following cell lines: NeHepLxHT cells, NeHepLxHT transfectants expressing of pre-S2Δ mutant surface proteins. The expression of APE1 was elevated in NeHepLxHT cells expressing hepatitis mutant surface proteins but not in control cells (Figure 5A). To confirm further that HBV large surface mutant proteins can induce APE1 * in vivo*, we created transgenic mice that express the pre-S2 deletion form of HBV large surface protein under the control of its native promoter. The expression of HBV large surface protein was detected in the liver (Figure 5B). Elevated expression of APE1 was observed in liver tissue in pre-S2Δ transgenic mice. As shown in Figure 5C, expression of APE1 in transgenic mice liver tissues were quantified.

### 2.6. Increased APE1 Expression Is Observed in ER Stress-Associated Liver Tumor Tissues

**Figure 6:**
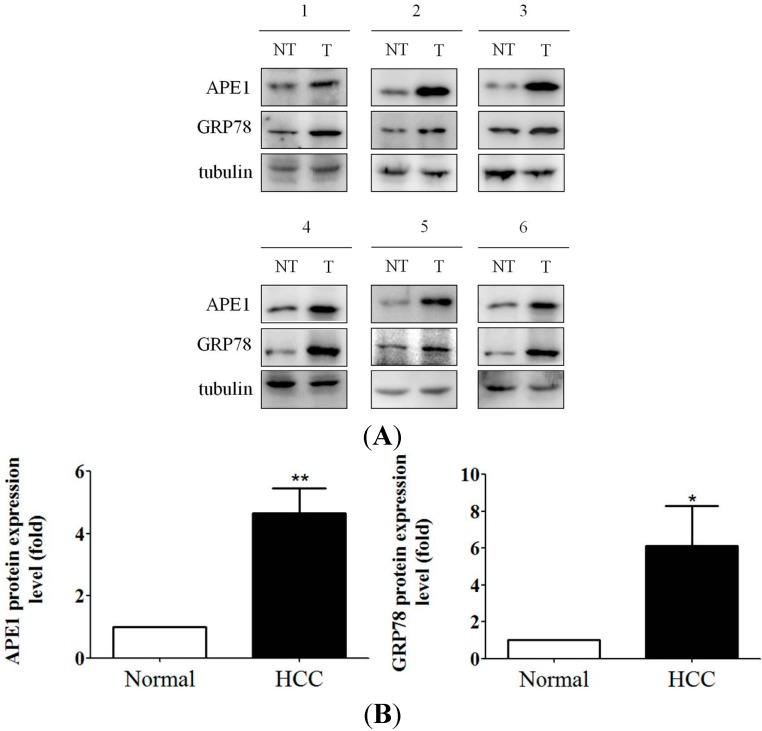
Highly APE1 expression was observed in ER stress-correlated human liver tumor tissue. (**A**) Increased APE1 is associated with high GRP78 expression in human liver tumor tissues *in vivo*. The expression of APE1 and GRP78 in control liver tissue and tumor tissue were determined by Western blotting for six individual samples; (**B**) The expression levels of APE1 and GRP78 were quantified by using ImageJ software. The data represent the mean of APE1 protein expression level from three independent experiments. Columns, mean; bars, SD (*n* = 3). Significant differences (*, *p* < 0.05 and **, *p* < 0.01) between the control and experimental group are marked with an asterisk.

In addition, human liver tumor samples have been used to quantify APE1 and GRP78 expression. First of all, expression of GRP78 was determined in several human liver samples by using western blotting. Next, human liver tumor samples with high GRP78 expression were then used to analyze APE1 expression. High expression of APE1 proteins were observed in hepatocytes that express high GRP78 proteins (Figure 6A). Furthermore, as indicated in Figure 6B, expression levels of APE1 and GRP78 were quantified. For example, the average APE1 and GRP78 expression levels in six human liver tumor tissues were enhanced to about 4.7- and 6.1-fold compared with human normal liver tissues. These results altogether demonstrate that pre-S mutant surface proteins or ER stress inducer can induce APE1, and the induction is possibly mediated through ER stress, at least partly.

## 3. Discussion

In this study, we show that ER stress enhances *APE1* gene expression by ER stress inducer in human hepatoma cancer cells, and that the induction of APE1 expression was at the transcriptional level. We also show that nuclear localization of APE1 is induced in response to ER stress. Furthermore, expression of APE1 was increased by pre-S2∆ proteins in human immortalized hepatocyte cells and pre-S2∆ transgenic mice liver tissue. In addition, ER stress and APE1 expression were also evaluated in human liver tumor samples. The result indicated that APE1 expression was correlated with ER stress in human tumor samples. Overall, our data provides evidence for a relationship between APE1 and ER stress, and suggests that regulation of APE1 expression by the mechanisms of ER stress signal pathways in ER stress-related hepatoma or pre-S2∆-containing tumor cells should be considered during chemotherapy in the future.

APE1 is an essential enzyme for the repair of DNA caused by oxidation and alkylation damage and thus protects cells against chemotherapeutic agents. Importantly, APE1 functions as a redox factor maintaining transcription factors in an active reduced state. Moreover, several studies demonstrated that APE1 was over-expressed in several human tumors and increased APE1 expression had been shown to be associated with poor prognosis [40,41,42]. For example, down-regulation of APE1 expression by siRNA significantly enhanced sensitivity of A549 cells to cisplatin [24]. Furthermore, knock down by siRNA or by pharmacological down regulation of Apel in glioma cells resulted in decreased radioresistance [43]. In addition, reduction of APE1 expression by siRNA in human osteogenic sarcoma cells led to increase sensitivity of osteosarcoma to DNA damaging agents: methyl methanesulfonate, H_2_O_2_, ionizing radiation, and chemotherapeutic agents [44]. A recent study also indicated that combined use of APE1 siRNA enhances the therapeutic efficacy of adenoviral-mediated *p53* gene transfer in hepatoma cells *in vitro* and *in vivo* [45]. Therefore, the expression level of APE1 is an important determinant of drug-induced apoptosis thereby modulating resistance to chemotherapy in cancer cells.

Our previous studies indicate that over-expression of pre-S2 large surface proteins have been demonstrated in the induction of endoplasmic reticulum (ER) stress [37], oxidative stress and DNA damage [46], COX-2 expression [35], cyclin A expression [47], degradation of p27Kip1 [48], vascular endothelial growth factor-A [49], interaction with α-acid glucosidase [50], lipid up-regulation [51], and Bcl-2 expression [52]. These results suggested that expression of HBV large surface proteins, especially pre-S2Δ mutant, might be important for hepatocarcinogenesis. For patients with cancer who are from HBV-endemic areas, routine screening for HBsAg before cytotoxic chemotherapy should be performed, and there was a trend of poorer survival for patients who had developed severe hepatitis during chemotherapy [53]. Furthermore, recent studies indicated that a pre-chemotherapy for high HBV viral levels is associated with poorer survival in HCC patients with chronic HBV infection [36], and the occurrences of HBV pre-S2Δ large surface proteins in HBV cancer patients are about 30% in Taiwan [37].

ER stress has been observed in many tumors, and ER stress is induced by many factors in the tumor microenvironment such as low glucose and hypoxia [54,55]. However, the role of ER stress in tumor development is still unclear. Previous studies have shown that many genes are involved in cell death or survival during ER stress, and three phases of adaptation, alarm, and apoptosis mechanisms are involved in regulating prolonged ER stress [56]. Severe ER stress induces activation of unique pathways that lead to cell death through apoptosis. Many studies have shown that multiple pathways are involved in ER stress-induced apoptosis, such as caspase12, PERk-eIF2α, IRE-1-JNK, and p53 pathways. On the other hand, many studies have also indicated that some genes participate in cell survival in response to ER stress. For example, over-expression of GRP78 in tumor cells has been linked to the progression of many human cancers [57,58]. The event of GRP78 over-expression in cancer cells causes resistance to chemotherapy. In addition, from our previous study, regulation of COX-2 expression is induced by ER stress in liver cancer cells [35], and over-expression of COX-2 is also observed in human cancer such HCC and colon cancer. Enhanced COX-2 expression is sufficient to induce mammary gland tumorigenesis in transgenic mice. However, COX-2 inhibitors suppress carcinogen-induced tumorigenesis in animal models [59,60]. Therefore, the degree of endoplasmic reticulum stress may play an important role in regulation of cell death or survival.

In summary, ER stress causes cell protection by increasing the expression of APE1. Furthermore, the event of APE1 overexpression was observed in ER stress-correlated HBV mutant protein, pre-S2Δ. These results may provide an important therapeutic strategy for chemotherapeutic drugs on ER stress-associated signaling pathways.

## 4. Experimental Section

### 4.1. Cell Culture and Material

Huh-7 and HepG2 cell lines were obtained from ATCC (Manasses, VA, USA). These cell lines were maintained at 37 °C in a 5% CO_2_ atmosphere in DMEM supplemented with 10% heat-inactivated fetal bovine serum, 100 units/mL penicillin, and 100 μg/mL streptomycin (Invitrogen, Ground Island, NY, USA). Culture medium was replaced every 2 days. ECL Western blot detection system was purchased from GE Healthcare (Pittsburgh, PA, USA). Anti-GRP78 was purchased from Transduction Laboratories. Anti-APE1, and β-actin antibodies were obtained from Santa Cruz Biotechnology (Santa Cruz, CA, USA). Anti-p-Ser^276^-p65, anti-rabbit IgG-horseradish peroxidase (HRP) conjugates, and rabbit anti-mouse IgG-HRP conjugates antibodies were purchased from Cell Signaling (Beverly, MA, USA). Anti-HA antibody was purchased from Calbiochem (San Diego, CA, USA).

### 4.2. Western Blot Analysis

The cell lysates were collected with RIPA lysis buffer (50 mM Tris-Cl pH 7.4. 150 mM NaCl. 1% NP40. 0.25% Na-deoxycholate. 1 mM PMSF, 1 mM EDTA; 5 μg/mL Aprotinin.) containing protease inhibitors (1 mM PMSF, 1 mM orthovanadate, 1 mM EDTA, and 10 μg/mL Leupeptin). NeHepLxHT and NeHepLxHT cell lysates were gift from Lily Hui-Ching Wang. Lysates from human normal and tumor samples were provided by Ih-Jen Su. Human liver tissue samples were collected from the period 1995 to 2002, and it was not required to apply the institutional review board (IRB) guidelines during this period. Protein concentrations of cell lysates were measured using a Micro BCA protein assay reagent kit (Pierce, Rockford, IL, USA). The cell lysates were subjected to SDS-PAGE, and transferred to nitrocellulose membranes (Millipore, Bedford, MA, USA.). The membranes were blocked with 5% (*w*/*v*) non-fat milk in PBS containing 0.1% Tween-20. After blocking with TBST containing 5% nonfat milk for 1 h, the membranes were probed with the following antibodies against: HA, β-actin, GRP78, and APE1 antibodies in 1% TBST nonfat milk at 4 °C overnight. The membrane was washed thrice with TBST for a total of 15 min. The secondary anti-mouse IgG-HRP conjugates or anti-rabbit IgG-HRP conjugates (1:2000 dilutions) was subsequently incubated with the membrane for 1 h at room temperature and was washed extensively for 50 min with TBST. The blots were visualized with the enhanced chemiluminescence (GE, Pittsburgh, PA, USA), and according to the manufacturer’s instructions. The blots were developed with the ECL-Western blot detection system according to the manufacturer’s instruction.

### 4.3. MTT Assay

Cell viability was assessed using the MTT assay in three replicates. HepG2 and APE1 shRNA transfectant cells were seeded at 1 × 10^4^ per well in 24-well flat-bottomed plates and incubated in 10% FBS-supplemented DMEM for 24 h. Cells were treated with 2.5 μg/mL tunicamycin in the same medium. Controls received the DMSO vehicle at a concentration same as that in drug-treated cells. After 2 days, the drug-containing medium was replaced with 200 μL of 10% FBS-supplemented DMEM containing 0.5 mg/mL MTT, and cells were incubated in the CO_2_ incubator at 37 °C for 4 h. Medium was removed, the reduced MTT was solubilized in 600 μL of DMSO per well, and 100 μL aliquots from each well were transferred to 96-well plates to measure absorbance at 570 nm.

### 4.4. Real-Time PCR

After treatment, the cells were washed with cold PBS and then cells were harvested. Total RNA was extracted from HepG2 cells using TRIzol reagent (Life Technologies, Grand Island, NY, USA) and chloroform extraction. RT was performed using 2 μg of total RNA. The cDNA synthesis was performed using 200 U of Moloney murine leukemia virus reverse transcriptase, 5 μM oligoDT, 1 mM dNTP solution, and 3 mM Mg^2+^ in a volume of 20 μL. In real-time PCR, PCR was performed for the resulting RT products using oligonucleotide primers specific for APE1, and GAPDH. The primers used were as follows: *APE1* gene (F) 5'-AGG CGA TGA GGA GCA TGA TC-3'/(R) 5'-CAG ACC TCG GCC TGC ATT AG-3'; *GAPDH* gene (F) 5'-GATTCCACCCATGGCAAATTC-3'/(R) 5'-AGCATCGCCCCACTTGATT-3'. All PCR reactions were performed with ABI 7500 Fast Real-Time PCR System using DNA-binding SYBR Green dye for detection of the PCR products and results were analyzed by ABI StepOne Software version 2 (Life technologies, Grand Island, NY, USA).

### 4.5. Transfection of pLKO-APE1 shRNA Vector

The pLKO shRNA vectors used for knockdown of APE1 is the following: TRCN0000007959 (APEX1). The pLKO and pLKO-APE1 vectors were obtained from National RNAi Core Facility (Taipei, Taiwan). HepG2 cells were transfected with pLKO or pLKO-APE1 shRNA plasmids by using Invitrogen LipofectAMINE 2000 reagent according to the manufacturer’s protocol. Cells were then selected by puromycin for 1 week. The pLKO and pLKO-APE1 shRNA stable clone cell lines were established by western blotting.

### 4.6. Histological Analysis of Pre-S2Δ Transgenic Mice Liver Tissue

The transgenic mouse liver tissues were gifts from Ih-Jen Su. The pre-S2Δ transgenic mice were constructed by injection of pre-S2Δ gene fragment into the male pronucleus of fertilized mouse ova. Microinjection was performed in Fvb/n mice. After 12 months, liver tissue from Pre-S2Δ transgenic mice were sacrificed, and their liver tissues were removed and cryosectioned (5 μm). Cryosections were fixed with 3.7% formaldehyde and acetone. Endogenous peroxidase was removed with 3% hydrogen peroxide. The cryosections were washed with PBS three times and incubated with anti-APE1 or anti-preS (gift from Lily Hui-Ching Wang) antibodies overnight at 4 °C. After subsequent reaction with peroxidase-conjugated secondary antibody, an aminoethyl carbazole substrate kit (Zymed Laboratories, San Francisco, CA, USA) was used for color development.

### 4.7. Statistical Analysis

Results were presented as the mean ± SD, and statistical comparisons were made using the Student’s *t* test. Statistically significant difference from control was defined at * *p* < 0.05 and ** *p* < 0.01, respectively.

## 5. Conclusions

In conclusion, ER stress is an inducer for APE1 expression in human cancer cells. Induced expression of APE1 and GRP78 was increased in HepG2 and Huh-7 cells by two ER stress inducers, tunicamycin and brefeldin A. Downregulation of APE1 expression by APE1 shRNA decreased cell survival rate during ER stress. In addition, hepatitis B virus pre-S2∆ large mutant surface protein, an ER stress-induced protein, also increased GRP78 and APE1 expression in the normal hepatocyte NeHepLxHT cell line. Induction of APE1 expression was observed in ER stress-correlated liver tumor samples. Our results have improved our understanding of the relationship between ER stress and APE1 expression. We believe that our findings provide a foundation for performing further studies in the future, especially in liver cancer cells.
